# Description of Labrys sedimenti sp. nov., isolated from a diclofenac-degrading enrichment culture, and genome-based reclassification of Labrys portucalensis as a later heterotypic synonym of Labrys neptuniae

**DOI:** 10.1099/ijsem.0.006778

**Published:** 2025-05-08

**Authors:** Andrea Csépányi, András Táncsics, Márton Pápai, Erzsébet Baka, Erika Tóth, Károly Bóka, Hussein Daood, István Szabó, Balázs Kriszt

**Affiliations:** 1 Department of Molecular Ecology, Institute of Aquaculture and Environmental Safety, Hungarian University of Agriculture and Life Sciences, Gödöllő, Hungary; 2 Department of Microbiology, Eötvös Loránd University, Budapest, Hungary; 3 Department of Plant Anatomy, Eötvös Loránd University, Budapest, Hungary; 4 Laboratories of Food Analysis, Institute of Horticultural Sciences, Hungarian University of Agriculture and Life Sciences, Gödöllő, Hungary; 5 Department of Environmental Toxicology, Institute of Aquaculture and Environmental Safety, Hungarian University of Agriculture and Life Sciences, Gödöllő, Hungary; 6 Department of Environmental Safety, Institute of Aquaculture and Environmental Safety, Hungarian University of Agriculture and Life Sciences, Gödöllő, Hungary

**Keywords:** biodegradation, catechol 1,2-dioxygenase, diclofenac, *Labrys*

## Abstract

A Gram-stain-negative strain, designated as Zidic-5^T^, was isolated from diclofenac-degrading enrichment culture and characterized using a polyphasic approach to determine its taxonomic position. The 16S rRNA gene sequence analysis revealed that strain Zidic-5^T^ belongs to the genus *Labrys*, with the highest 16S rRNA gene similarity to *Labrys neptuniae* LMG 23578^T^ (99.13%), followed by *Labrys portucalensis* F11^T^ (99.06%), *Labrys methylaminiphilus* JLW10^T^ (98.58%) and *Labrys okinawensis* DSM 18385^T^ (98.32%). The draft genome sequence of strain Zidic-5^T^ is 7.64 Mb long, and the G+C content of the genome is 63.5 mol%. The orthologous average nucleotide identity and digital DNA–DNA hybridization relatedness values between strain Zidic-5^T^ and its closest relatives were below the threshold values for species demarcation, confirming that strain Zidic-5^T^ is distinctly separated from its closest relatives. Additionally, comparative whole-genome analysis of type strains of *L. neptuniae* and *L. portucalensis* indicated that they belong to the same genomic species, suggesting that *L. portucalensis* is a later heterotypic synonym of *L. neptuniae*. Cells of strain Zidic-5^T^ were strictly aerobic, coccoid-shaped and non-motile. The predominant fatty acids (>10% of the total) of strain Zidic-5^T^ were C_18 : 1_ ω7c, C_16 : 0_ and C_19 : 0_ cyclo ω7c. The major ubiquinone of strain Zidic-5^T^ was Q-10, while the major polar lipids were phosphatidylcholine, diphosphatidylglycerol, phosphatidylglycerol, phosphatidylethanolamine and aminophospholipid. Based on the polyphasic study, it is concluded that strain Zidic-5^T^ represents a novel species of the genus *Labrys*; thus, the name of *Labrys sedimenti* sp. nov. is proposed. The type strain of the species is strain Zidic-5^T^ (=LMG 33565^T^=NCAIM B.02686^T^).

## Introduction

The genus *Labrys* was first described by Vasilyeva and Semenov [1] in a study that reported on the isolation of a new budding prosthecate bacterium. Description of the genus was later emended by Islam *et al*. [2] and Albert *et al*. [3]. It is affiliated with the family *Xanthobacteriaceae* (class *Alphaproteobacteria*) and currently contains eight validly described species, which were isolated from various environments, including rhizosphere soil, freshwater lake, contaminated sediment and root nodules [2,7]. Members of the genus are aerobes or facultative anaerobes [3], and cells are mostly rod-shaped, but some species have cells with triangular symmetry and motile or non-motile [2]. The major cellular fatty acids are C_19:0_ cyclo *ω*8*c*, C_16: 0_, C_18 :0_ and C_18 :1_ *ω*7*c*, and the G+C content of the DNA varies between 61.0 and 66.0 mol% [2]. The predominant quinone is ubiquinone-10 (Q-10) [2].

The most deeply studied member of the genus is *Labrys portucalensis* F11^T^ due to its ability to degrade a wide variety of xenobiotics, including pharmaceutical compounds. Initially, it was described as a fluorobenzene-degrading bacterium [6], but later its ability to degrade diclofenac, carbamazepine, fluoxetine and fluoroquinolone antibiotics was also shown [8]. The ibuprofen-degrading ability has been recently shown in the case of an *Labrys neptuniae* strain, isolated from sewage sludge [9].

The present study aimed to perform the polyphasic, formal description of a bacterial strain, designated as Zidic-5^T^. The strain was isolated from a diclofenac-degrading enrichment culture and initially identified as a member of the genus *Labrys* (*Xanthobacteraceae*).

## Methods

### Enrichment, isolation and growth conditions of bacterial strains

In this study, a novel strain belonging to the genus *Labrys* was isolated from a diclofenac-degrading enrichment culture, inoculated with a sediment sample of the River Zagyva (Hungary), in the close vicinity of the catchment area of a communal wastewater treatment plant. The sediment sample was collected in May 2022 and transported to the laboratory immediately. Selective enrichment of diclofenac-degrading bacterial communities was carried out as described earlier [10]. At the end of the enrichment period, equal volumes of the triplicate enrichments were pooled, and then 1.0 ml of the pooled enrichment medium was serially diluted in physiological salt solution (0.9% NaCl). Subsequently, 100 µl from each dilution was spread onto R2A media agar plates, which were incubated at 28 °C for up to 1 week. Distinct bacterial colonies were inoculated onto new R2A agar plates using a streak plating technique and incubated at 28 °C for 72 h. Strains were maintained on R2A agar plates at 4 °C and stored long term at −80 °C in a glycerol-R2A solution (30% v/v).

### Bacterial community analysis of the enrichments

The taxonomic composition of the enrichment bacterial communities was revealed by 16S rRNA gene amplicon sequencing as described earlier [11]. Paired-end fragment reads were generated on an Illumina MiSeq sequencer using MiSeq Reagent Kit v3 (600-cycle) by SeqOmics Biotechnology Ltd. (Mórahalom, Hungary). The raw sequencing data were analysed according to Banerjee *et al*. [12] to cluster sequences into operational taxonomic units (OTUs) and calculate the diversity of the samples using rarefaction curves. Read numbers were between 40,000 and 50,000 during the data processing performed by the MiSeq SOP of mothur v1.41.1 [13,14]. Sequence reads were deposited in Sequence Read Archive of the National Center for Biotechnology Information (NCBI SRA) under BioProject ID PRJNA1050906.

### Phylogenetic analysis

Genomic DNA of isolate Zidic-5^T^ was extracted by using the DNeasy® UltraClean® Microbial DNA isolation Kit (Qiagen, Germany). Subsequently, the partial 16S rRNA gene was PCR amplified by using primers 27F and 1492R and Sanger-sequenced as reported earlier [12]. Sequencing products were separated on a Model 3130 Genetic Analyzer (Applied Biosystems) as described earlier [12]. The PCR amplified 1,399 bp long 16S rRNA gene sequence of strain Zidic-5^T^ was compared with 16S rRNA gene sequences using the EzBioCloud server (http://www.ezbiocloud.net/eztaxon) to determine an approximate phylogenetic affiliation [15]. Multiple alignment of sequences (by clustal w with default parameters), calculation of evolutionary distance by Kimura’s two-parameter calculation model [16] and construction of a neighbour-joining, maximum likelihood and maximum parsimony phylogenetic trees were conducted using mega x, where the number of bootstrap replications was set to 1,000 [17,19]. The whole-genome sequencing of strains Zidic-5^T^, *L. neptuniae* LMG 23578^T^ and *L. portucalensis* LMG 23412^T^ was performed by SeqOmics Biotechnology Ltd. (Hungary) as described by Benedek *et al*. [20]. Briefly, the NEBNext Ultra II DNA Library Prep Kit (Illumina, USA) was used to generate *in vitro* fragment libraries according to the instructions of the manufacturer. Paired-end fragment reads were generated, and the primary data analysis was performed as described by Bedics *et al*. [21]. *De novo* assembly and scaffolding were performed with SPAdes version 3.13.0 [22]. The complete genome of the investigated strains was automatically annotated using the NCBI Procaryotic Genomes Automatic Annotation Pipeline v4.5 [23]. A UBCG (up-to-date bacterial core gene) phylogenetic tree was constructed by utilizing an up-to-date bacterial core gene set and a pipeline based on a concatenated alignment of 92 core genes [24]. The digital DNA–DNA hybridization (dDDH) values among strain Zidic-5^T^ and related species were determined using the Genome-to-Genome Distance Calculator (https:// ggdc. dsmz. de/) version 2.1 [25]. The GenBank database (www.ncbi.nlm.nih.gov/genbank) was applied to retrieve the available reference genomes for comparison purposes. The orthologous average nucleotide identity (OrthoANI) values between the strain Zidic-5^T^ and its closest relatives were calculated using the OAT software [26]. The initial whole-genome-based phylogenetic analysis was performed by the MiGA pipeline (http://microbial-genomes.org/) [27]. To identify gene clusters involved in the degradation of aromatic compounds, CLC Genomics Workbench Tool v21 (Qiagen) and BlastKOALA (version 3.0) were used [28].

### Phenotypic and biochemical characterization

Gram staining was performed according to Claus [29]. Cell size, morphology and motility of strain Zidic-5^T^ were studied by transmission electron microscopy described by Szoboszlay *et al*. [30]. Physiological and biochemical tests were examined according to the protocols of Barrow and Feltham [31] and Smibert and Krieg [32]. The temperature and pH growth tests were performed in nutrient broth (DSM medium no. 1) that was adjusted with sterile, 20% (m/v) KOH or HCl solutions to the corresponding pH. The temperature growth test was carried out between 4 and 45 °C and pH between 3.0 and 12.0. The salt tolerance of the strain Zidic-5^T^ was determined by inoculating the strain into the nutrient broth supplemented with 0.1–5% (w/v) NaCl. The growth of strain at different temperatures, pH and salt concentration was determined based on the absorbance (600 nm) of the R2A broth. Other biochemical characteristics and enzyme activities of the strain Zidic-5^T^ were investigated by using API 50 CH and API ZYM test kits (bioMérieux, France) according to the manufacturer’s instructions. Anaerobic growth was tested in nutrient broth with and without the addition of 0.15% (w/v) KNO_3_ at 28 °C similarly as it was described by Farkas *et al*. [33]. The diclofenac degradation ability of strain Zidic-5^T^ was investigated by HPLC measurements according to Pápai *et al*. [10]. The two closest relative type strains, *L. portucalensis* LMG 23412^T^ and *L. neptuniae* LMG 23578^T^, were used as positive controls during the degradation experiments.

### Chemotaxonomy

The whole-cell fatty acids, respiratory quinones and polar lipids were analysed by DSMZ Services, Leibniz-Institut DSMZ - Deutsche Sammlung von Mikroorganismen und Zellkulturen GmbH (Braunschweig, Germany). For the examinations, cells were cultivated on R2A medium at 28 °C for 48 h. After harvesting biomass, cellular fatty acids were converted into fatty acid methyl esters by saponification, methylation and extraction by applying the protocol of Sasser [34]. Subsequently, the fatty acid methyl ester mixtures were separated by gas chromatography and detected by a flame ionization detector. In subsequent analysis, fatty acids were identified by a GC-MS analysis, on an Agilent GC-MS 7000D system [35]. Thus, cellular fatty acids were identified based on both retention time and mass spectra. The polar lipids and respiratory quinones were extracted based on the protocol modified after Bligh and Dyer [36]. The separation and detection of the total lipid material and functional groups were performed as described by Banerjee *et al*. [12]. The quinones were analysed by LDC Analytical (Thermo Separation Products) HPLC apparatus fitted with a reverse-phase column (Macherey-Nagel; 2.125 mm, 3 µm, RP18) using methanol/heptane 9 : 1 (v/v) as eluant.

## Results and discussion

### Ecology and isolation of strain Zidic-5^T^

In all of the parallel diclofenac-degrading enrichment cultures, members of the genus *Pseudomonas* overwhelmingly dominated the bacterial communities (Fig. S1, available in the online Supplementary Material). Their relative abundance values varied between 37 and 50%. Other highly abundant community members were the genera *Afipia* (10–13 %), *Rhodanobacter* (4–10 %), *Castellaniella* (0.5–7.5 %), *Labrys* (0.8–8 %) and *Sphingopyxis* (1.5–4.5 %). Most of these genera have already been reported to take part in the degradation of pharmaceuticals, including diclofenac as well [8,10, 37, 38]. In total, 15 bacterial strains were isolated from the enrichments. One of the isolates, designated as Zidic-5^T^, proved to be a member of the genus *Labrys* after initial analysis of its 16S rRNA gene sequence. Subsequently, a complex polyphasic study was performed to assess its exact taxonomic affiliation.

### Phylogenetic analysis based on the 16S rRNA gene

**Fig. 1.**
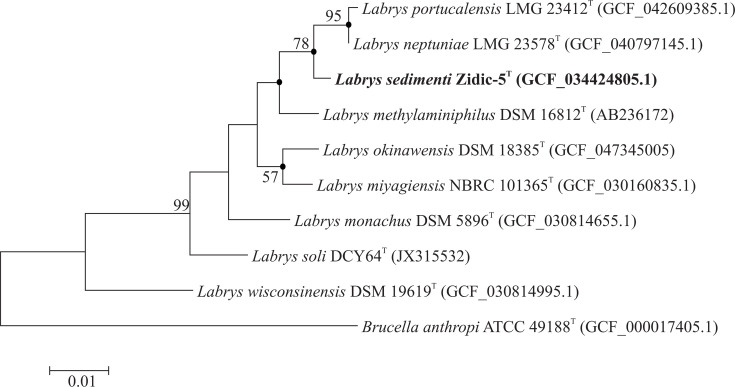
Maximum likelihood phylogenetic tree based on 16S rRNA gene sequences highlighting the phylogenetic position of *Labrys sedimenti* Zidic-5^T^ strain relative to other related taxa. Filled circles indicate that the corresponding nodes occurred with every tree-making algorithm used in the study. Bootstrap values (> 50 %) based on 1,000 bootstrap replicates are shown at branch nodes. *Brucella anthropi* NBRC 15819^T^ was used as an outgroup. Bar, 0.01 substitutions per nucleotide position.

Comparative 16S rRNA gene sequence analysis revealed that strain Zidic-5^T^ belonged to the genus *Labrys*, most closely related to *L. neptuniae* LMG 23578^T^ with a similarity of 99.13%, followed by *L. portucalensis* LMG 23412^T^, *Labrys methylaminiphilus* JLW10^T^ and *Labrys okinawensis* DSM 18385^T^ with a similarity of 99.06%, 98.58% and 98.32 %, respectively. Other type strains of the genus showed less than 98% 16S rRNA gene sequence similarity with strain Zidic-5^T^. On the other hand, the closest non-type strain relative of strain Zidic-5^T^ with a known whole-genome sequence was *Labrys* sp. strain WJW, sharing 99.71% 16S rRNA gene similarity. This latter strain was described as a graphene oxide-degrading bacterium, but its exact phylogenetic position was not investigated at that time [39]. The phylogenetic relationship between strain Zidic-5^T^ and the above-mentioned bacterial strains was also confirmed by the phylogenetic trees, and strain Zidic-5^T^ formed a separate lineage in the genus *Labrys* among its closest relatives (Figs 1 and S2). Furthermore, it was also observable that *L. neptuniae* and *L. portucalensis* clustered close together on the phylogenetic trees, which was because they shared 99.87% 16S rRNA gene sequence similarity.

### Genome features and phylogenomic analysis

**Fig. 2.**
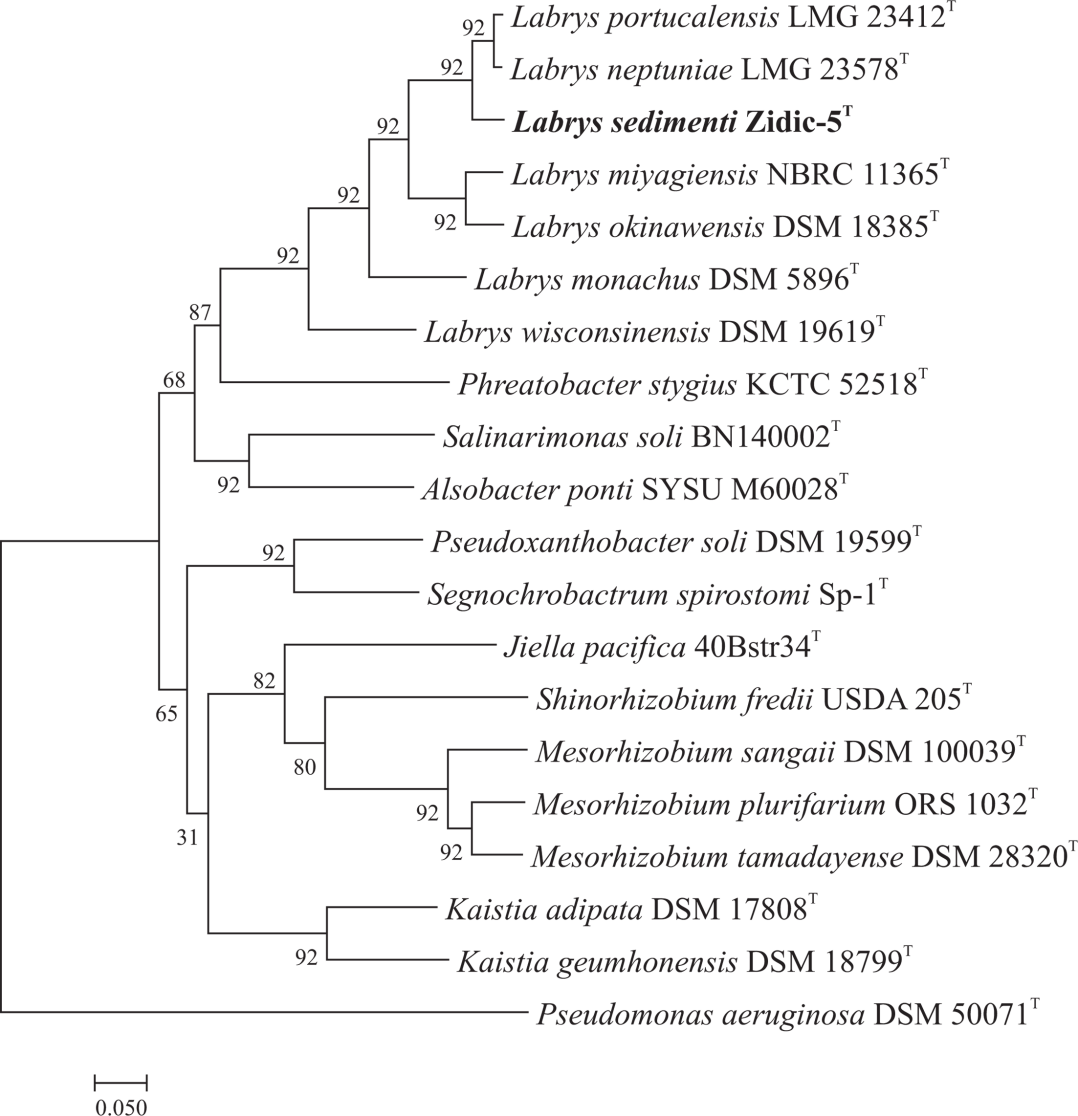
UBCG phylogenomic tree based on concatenated alignment of 92 core genes showing the phylogenetic position of *Labrys sedimenti* Zidic-5^T^ strain relative to other closely related species. Bar, 0.05 substitution per nucleotide position.

Whole-genome sequence of strain Zidic-5^T^ has been deposited at GenBank under the accession number JAXOUF000000000. The high-quality draft genome of strain Zidic-5^T^ had a total length of 7,643,142 bps with a G+C content of 63.5 mol%, which fell into the range of *Labrys* species. The genome sequence was assembled into 109 contigs with an N50 value of 368.6 kb and an average coverage value of 51. Since the whole-genome sequences of the closest relatives were not available during the study, for comparison purposes, whole genomes of *L. neptuniae* LMG 23578^T^ and *L. portucalensis* LMG 23412^T^ were sequenced (details are given in Table S1). The OrthoANI values between strain Zidic-5^T^ and *L. neptuniae* LMG 23578^T^ and *L. portucalensis* LMG 23412^T^ were 92.23% and 92.09 %, respectively. Other type species of the genus with known genome sequence showed less than 80% OrthoANI values when compared with strain Zidic-5^T^ (Fig. S3). Interestingly, the OrthoANI value between whole genomes of *L. neptuniae* LMG 23578^T^ and *L. portucalensis* LMG 23412^T^ was 97.98%, which is well above the 95–96 % threshold value used to delineate bacterial species. Accordingly, this result implied that *L. neptuniae* and *L. portucalensis* belong to the same genomic species. *In silico* dDDH relatedness values between strain Zidic-5^T^ and *L. neptuniae* LMG 23578^T^ and *L. portucalensis* LMG 23412^T^ were 46.4% and 46.1 %, respectively. This result also implied that strain Zidic-5^T^ can be delineated at the species level from its closest relatives. On the other hand, this value between *L. neptuniae* LMG 23578^T^ and *L. portucalensis* LMG 23412^T^ was 81.7%, well above the 70% threshold value defined for the species-level delineation. Regarding the non-type strain *Labrys* sp. WJW, it showed 96.6% OrthoANI and 71.9% dDDH relatedness with strain Zidic-5^T^, indicating that they belong to the same genomic species. The UBCG phylogenomic tree confirmed the distinct phylogenetic position of strain Zidic-5^T^, similarly to the 16S rRNA gene-based phylogenetic tree (Fig. 2).

### Functional genomics

BlastKOALA analysis predicted that the Zidic-5^T^ genome encodes 78 complete pathway modules organized into the following functional subcategories within the metabolism category: carbohydrate metabolism, energy metabolism, lipid metabolism, nucleotide metabolism, amino acid metabolism, metabolism of cofactors and vitamins, biosynthesis of terpenoids and polyketides, biosynthesis of other secondary metabolites and xenobiotics biodegradation. In the latter case, two complete pathways for aromatics degradation were found: the benzoate degradation pathway and the catechol *ortho*-cleavage pathway (Table S2). Regarding energy metabolism, the assimilatory nitrate and sulphate reduction pathways were found to be complete. On the other hand, pathogenicity and drug resistance-related gene sets (e.g. beta-lactam resistance and multidrug resistance efflux pump-related gene sets) were highly incomplete, implying the non-pathogenic nature of strain Zidic-5^T^, similarly to other members of the genus *Labrys*.

Previous studies have suggested that key enzymes of the beta-ketoadipate pathway, e.g. protocatechuate 3,4-dioxygenase and catechol 1,2-dioxygenase (C12O) which catalyse intradiol ring-cleavage, play a crucial role in the degradation of diclofenac [40,41]. Therefore, it was investigated whether the corresponding genes can be found in the genome of strain Zidic-5^T^ and the case of the closest relatives. In the case of strain Zidic-5^T^, one gene was found that encoded a C12O enzyme. This gene was part of a cluster that also encoded benzoate 1,2-dioxygenase, and enzymes responsible for the further processing of the *ortho*-cleavage products (e.g. muconate cycloisomerase and muconolactone isomerase). Similar gene clusters were observable in the genome of *L. portucalensis* LMG 23412^T^ and *L. neptuniae* LMG 23578^T^, respectively. In the case of the other, less closely related members of the genus *Labrys,* it was observable that the catechol *ortho*-cleavage pathway was either incomplete (*L. wisconsinensis* DSM 19619^T^, *L. monachus* DSM 5896^T^) or completely missing (*L. okinawensis* DSM 38385^T^ and *L. miyagiensis* NBRC 11635^T^). Nevertheless, in the case of *Raoultella* sp. KDF8 role of a quercetin 2,3-dioxygenase (an extradiol dioxygenase) was also suggested in the degradation of diclofenac [40], but such an enzyme was not coded in the genome of strain Zidic-5^T^ or genomes of its closest relatives.

### Phenotypic and biochemical characteristics

For the comparative phenotypic analyses, *L. neptuniae* LMG 23578^T^ and *L. portucalensis* LMG 23412^T^ were used as reference strains under the same laboratory conditions. Microscopic observations of the cells of strain Zidic-5^T^ revealed that it is Gram-staining-negative; cells are non-motile, capsulated and coccoid-shaped with a diameter of 0.8–1 µm with no flagellum (Fig. S4). However, dividing cells of Zidic-5^T^ may appear as rods. Colonies have a creamy-white colour, medium size and round shape after 2 days of incubation on R2A agar plates at 28 °C. Growth of the strain Zidic-5^T^ was observed between a temperature of 15–42 °C, in a pH range of 5–11, and could tolerate NaCl up to 3% (w/v). Optimal growth was observed at 25–33 °C, pH 6–8 and in the presence of a maximum of 1% (w/v) NaCl.

In the case of the API ZYM tests, positive reactions were observed for alkaline phosphatase, esterase (C4), leucine arylamidase and acid phosphatase. A weak positive reaction was recorded in the case of valine arylamidase. In the case of the reference strains, a highly similar pattern was observed, except *L. neptuniae* LMG 23578^T^, which was also positive for naphthol-AS-BI-phosphohydrolase. In the case of the API 50 CH tests, it was observed that strain Zidic-5^T^ was able to use a wide variety of carbohydrates, including l-arabinose, d-ribose, d-xylose, d-galactose, d-glucose, d-fructose, d-mannose, l-sorbose, dulcitol, inositol, d-mannitol, aesculin, d-cellobiose, d-melibiose, xylitol, gentiobiose, d-turanose, d-lyxose, d-tagatose, d-fucose, d-arabitol and potassium-gluconate. Results of the API 50 CH and API ZYM tests are summarized in Table S3.

Diclofenac degradation tests showed that strain Zidic-5^T^, similarly to *L. portucalensis* LMG 23412^T^ and *L. neptuniae* LMG 23578^T^, was able to completely deplete the pharmaceutical compound from the test solution in 168 h. Meanwhile, in the abiotic samples, no significant depletion of diclofenac was observed. In the biotic samples, the microbial biomass of strain Zidic-5^T^ was also checked for the presence of diclofenac, giving a negative result, which further confirmed the observation that strain Zidic-5^T^ can degrade diclofenac under the investigated circumstances.

The differences in phenotypic characteristics between strain Zidic-5^T^ and its closest relatives are summarized in Table 1. Strain Zidic-5^T^ could be distinguished from its closest relatives based on the following characteristics: negative for d-sorbitol and d-trehalose utilization in the API 50 CH test, the ability to grow above 37 °C, the ability to grow in the presence of 3% (w/v) NaCl and cells are coccoid-shaped.

**Table 1.** Major phenotypic and biochemical characteristics of 1, strain Zidic-5^T^; 2, *L. portucalensis* LMG 23412^T^; and 3, *L. neptuniae* LMG 23578^T^ +, Positive; −, negative; w, weak positive. All data are from the present study.

Characteristic	1	2	3
Colony colour	Creamy white	White	White
Temperature range (°C)	15–42	15–37	15–37
pH range	5–11	5–11	5–11
NaCl tolerance (% w/v)	3	2	2
Oxidase	+	+	−
**Activity of**(**API ZYM**)			
Naphthol-AS-BI-phosphohydrolase	−	−	+
**Acid production from (API 50CH**)			
d-Sorbitol	−	+	+
Trehalose	−	+	+
Erythritol	−	−	+
l-Rhamnose	−	−	+
Arbutin	−	−	+
Raffinose	−	−	+
Gentiobiose	+	−	+
Turanose	+	−	+
l-Arabitol	−	−	+

### Chemotaxonomic characteristics

The major fatty acids (>10%) of strain Zidic-5^T^ were C_18 : 1_* ω*7c, C_16 : 0_ and C_19  :  0_ cyclo ω7c, which are similar to the reference strains *L. neptuniae* LMG 23578^T^ and *L. portucalensis* LMG 23412^T^. This result supported the affiliation of Zidic-5^T^ with the genus *Labrys*, although some quantitative differences compared to the reference strains were observed (Table 2). Besides, it was also observable that *L. neptuniae* LMG 23578^T^ and *L. portucalensis* LMG 23412^T^ showed almost identical fatty acid composition, further confirming that they belong to the same species. In the case of strain Zidic-5^T^, the major respiratory quinone was ubiquinone-10 (Q-10) (99.2%), while ubiquinone-9 (Q-9) was detected as a minor component (0.8%). The detected polar lipids were phosphatidylcholine (PC), diphosphatidylglycerol, phosphatidylglycerol (PG), phosphatidylethanolamine (PE), aminolipid, two aminophospholipids (APL) and two phospholipids (Fig. S5).

**Table 2.** Cellular fatty acid compositions of strain Zidic-5^T^ and related species obtained by GC-MS analysis Taxa: 1, strain Zidic-5^T^; 2, *L. portucalensis* LMG 23412^T^; 3, *L. neptuniae* LMG 23578^T^. Data are expressed as percentages of total fatty acids. Fatty acids which were lower than 1.0% in all strains are not shown. nd, not detected. tr, trace amount (< 1 %). All data are from the present study.

Fatty acid	1	2	3
**Saturated**			
C_16 : 0_	16.5	17.7	15.5
C_18 : 0_	4.0	2.0	2.9
**Unsaturated**			
C_18 : 1_* ω*7c	60.0	45.0	47.7
**Hydroxylated**			
C_14 : 0_ 3-OH	2.4	3.1	2.0
C_18 : 0_ 3-OH	1.1	nd	tr
**Methylated**			
C_18 : 1_ ω7c 11-methyl	1.4	1.1	tr
**Cyclic**			
C_19 : 0_ cyclo ω7c	13.0	27.8	28.4

## Conclusions

In conclusion, the results of the phenotypic, genomic and chemotaxonomic analyses showed that strain Zidic-5ᵀ represents a novel species within the genus *Labrys*. For this species, we propose the name of *Labrys sedimenti* sp. nov. Besides, results provided evidence to conclude that *L. portucalensis* LMG 23412^T^ and *L. neptuniae* LMG 23578^T^ belong to the same genomic species. Therefore, based on the principle of priority, we propose that *L. portucalensis* Carvalho *et al.* 2008 is a later heterotypic synonym of *L. neptuniae* Chou *et al.* 2007.

## Description of *Labrys sedimenti* sp. nov.

*Labrys sedimenti* (se.di.men’ti. L. gen. n. *sedimenti*, of sediment, referring to the sediment of the river Zagyva, Hungary, where the type strain was isolated).

Cells are Gram-stain-negative, strictly aerobic, coccoid-shaped, capsulated and non-motile. Can grow at temperatures ranging from 15 to 42 °C, in the presence of 0–3% (w/v) NaCl and at pH values of 5–11. Colonies are creamy-white coloured with entire margins. Positive for oxidase, catalase, urease and phosphatase activities. Can assimilate l-arabinose, d-ribose, d-xylose, d-galactose, d-glucose, d-fructose, d-mannose, l-sorbose, dulcitol, inositol, d-mannitol, aesculin, d-cellobiose, d-melibiose, xylitol, gentiobiose, d-turanose, d-lyxose, d-tagatose, d-fucose, d-arabitol and potassium-gluconate. The major cellular fatty acids are C_18 : 1_* ω*7c, C_16 : 0_ and C_19  :  0_ cyclo ω7c. Ubiquinone-10 (Q-10) is the major respiratory quinone. The major polar lipids are PC, PG, PE and APL. The DNA G+C content is 63.5 mol%.

The GenBank/EMBL/DDBJ accession numbers for the 16S rRNA gene sequence and whole-genome sequence of strain Zidic-5^T^ are PP318614 and JAXOUF000000000, respectively.

The type strain, Zidic-5^T^ (=LMG 33565^T^=NCAIM B.02686^T^), was isolated from a diclofenac-degrading enrichment culture inoculated with a sediment sample of the River Zagyva (Hungary).

## Supplementary material

10.1099/ijsem.0.006778 Uncited Supplementary Material 1.

## Abbreviations

AL: aminolipid
dDDH: digital DNA–DNA hybridization
NCBI SRA: Sequence Read Archive of the National Center for Biotechnology Information
OrthoANI: orthologous average nucleotide identity
OTU: operational taxonomic unit
PC: phosphatidylcholine
PE: phosphatidylethanolamine
PG: phosphatidylglycerol
UBCG: up-to-date bacterial core gene
