# Supplementary material for: Description of Labrys sedimenti sp. nov., isolated from a diclofenac-degrading enrichment culture, and genome-based reclassification of Labrys portucalensis as a later heterotypic synonym of Labrys neptuniae
Source: Int J Syst Evol Microbiol. 2025 May 8;75(5):006778. doi: 10.1099/ijsem.0.006778 (PMC12062540; doi:10.1099/ijsem.0.006778)
Supplement: Uncited Supplementary Material 1. [file ijsem-75-06778-s001.pdf]

Description of *Labrys sedimenti* sp. nov., isolated from a diclofenac-degrading enrichment culture, and genome-based reclassification of *Labrys portucalensis* as a later heterotypic synonym of *Labrys neptuniae*

Andrea Csépányi[1], András Táncsics[1], Márton Pápai[1], Erzsébet Baka[1], Erika Tóth[2], Károly Bóka[3], Hussein Daood[4], István Szabó[5], Balázs Kriszt[6]

[1]Department of Molecular Ecology, Institute of Aquaculture and Environmental Safety, Hungarian University of Agriculture and Life Sciences, Gödöllő, Hungary;

[2]Department of Microbiology, Eötvös Loránd University, Budapest, Hungary;

[3]Department of Plant Anatomy, Eötvös Loránd University, Budapest, Hungary

[4]Laboratories of Food Analysis, Institute of Horticultural Sciences, Hungarian University of Agriculture and Life Sciences, Gödöllő, Hungary

[5]Department of Environmental Toxicology, Institute of Aquaculture and Environmental Safety, Hungarian University of Agriculture and Life Sciences, Gödöllő, Hungary

[6]Department of Environmental Safety, Institute of Aquaculture and Environmental Safety, Hungarian University of Agriculture and Life Sciences, Gödöllő, Hungary

Corresponding author

András Táncsics, Department of Molecular Ecology, Institute of Aquaculture and Environmental Safety, Hungarian University of Agriculture and Life Sciences, Páter K. u. 1., H-2100 Gödöllő, Hungary. E-mail: tancsics.andras@uni-mate.hu

Supplementary material for publication in IJSEM online.

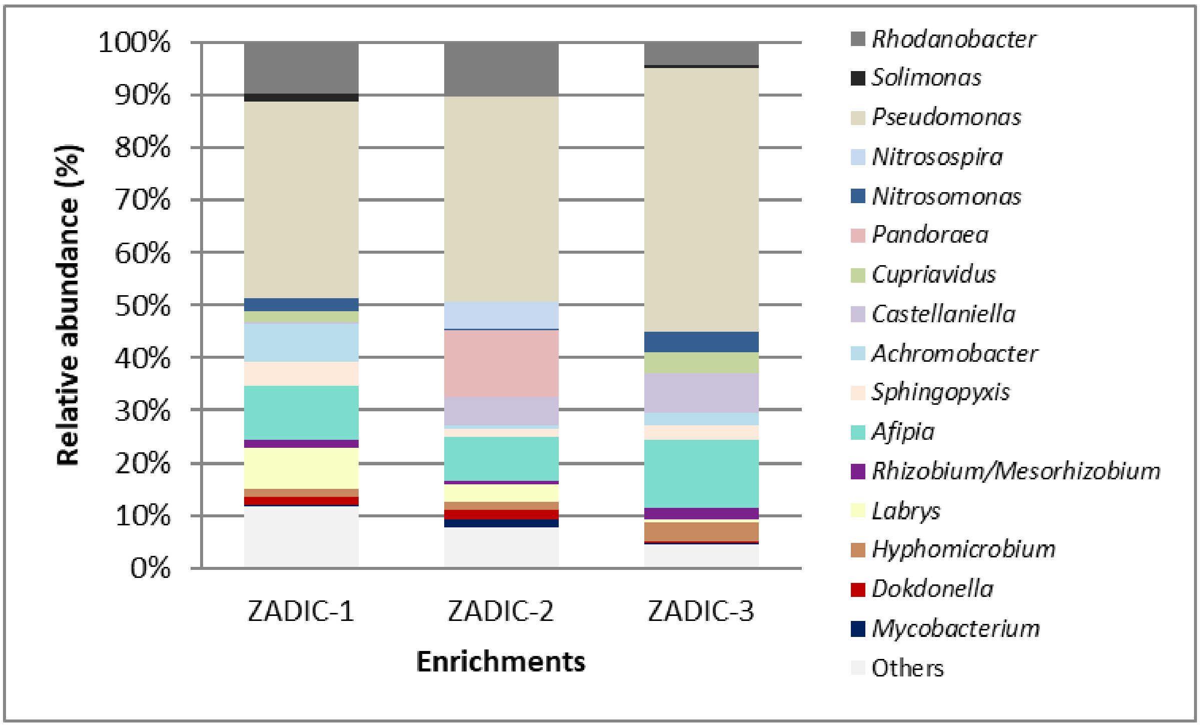

Figure S1. Genus level bacterial community structure of the diclofenac-degrading enrichment cultures as revealed by Illumina paired-end 16S rRNA gene amplicon sequencing. Only taxa contributing more than 1% abundance are depicted

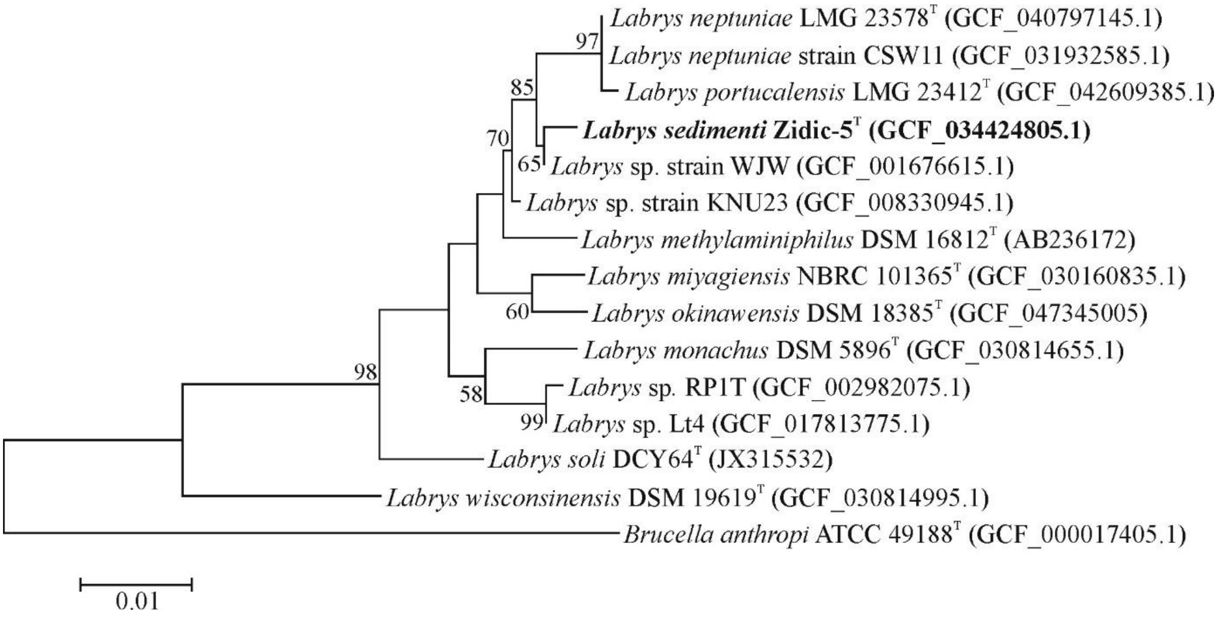

Figure S2. Maximum-likelihood phylogenetic tree based on 16S rRNA gene sequences highlighting the phylogenetic position of *Labrys sedimenti* Zidic-5^T strain relative to other members of the genus *Labrys*. The tree includes those non-type strains, in which case the whole-genome sequence is available. Bootstrap values (> 50%) based on 1000 bootstrap replicates are shown at branch nodes. Whole-genome sequence accession numbers are given in parenthesis. *Brucella anthropi* NBRC 15819^T was used as an out-group. Bar, 0.01 substitutions per nucleotide position.

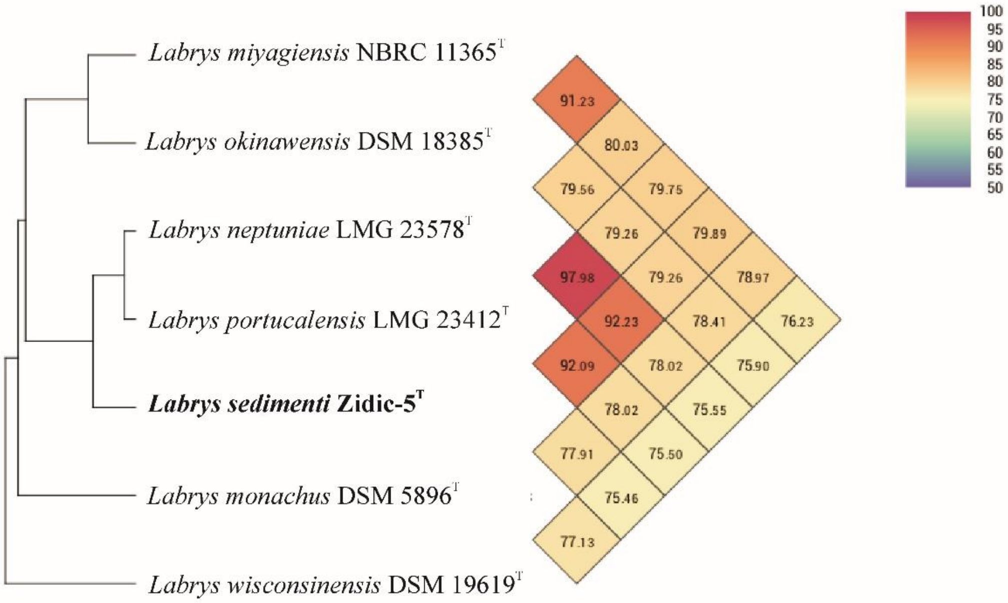

Figure S3. Heatmap generated with OrthoANI values calculated using the OAT software between strain Zidic-5^T and other closely related *Labrys* species. The color code indicates the closest species in red to the farthest in blue.

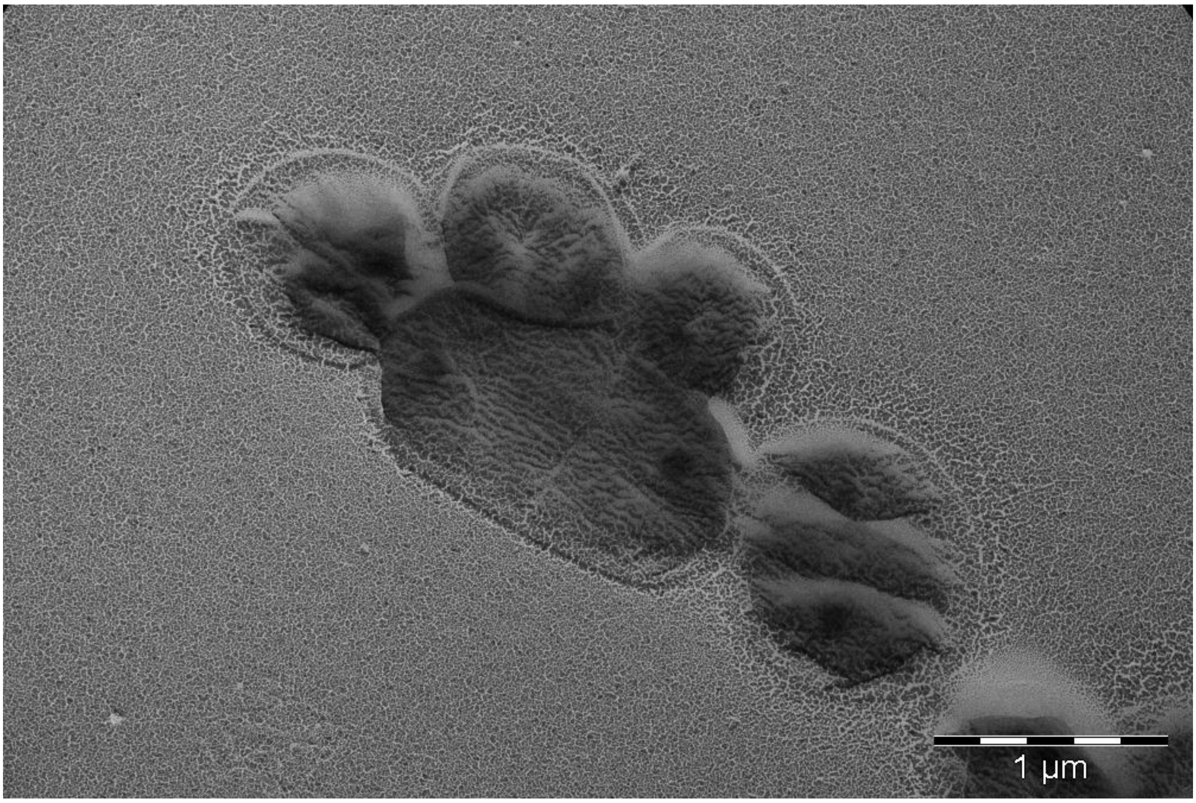

Figure S4. Transmission electron microscopic image of Zidic-5^T, grown on nutrient agar at 28ºC for 2 days. Scale Bar: 1 µm.

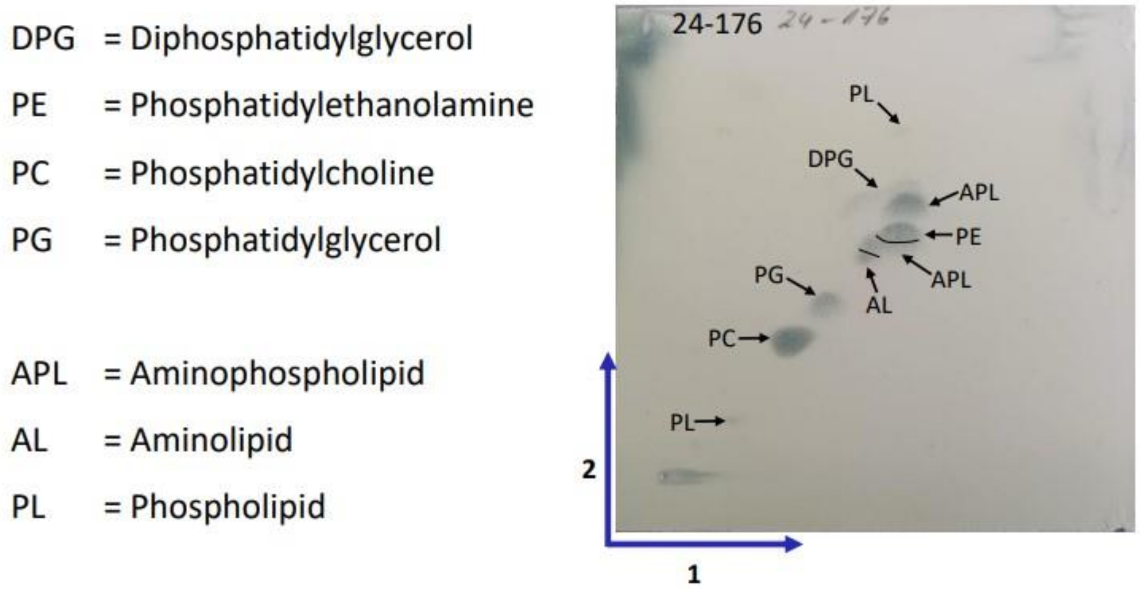

Figure S5. Two-dimensional thin layer chromatography (TLC) profile of the polar lipids of strain Zidic-5^{T}.

Table S1. Basic characteristics of the *Labrys* whole-genome sequences obtained in the present study. 1, strain Zidic-5^T; 2, *Labrys neptuniae* LMG 23578^T; 3, *Labrys portucalensis* LMG 23412^T

**Characteristics**	1	**2**	**3**
GenBank accession	JAXOUF000000000	JBFNQD000000000	JBHGPK000000000
No. of contigs	109	49	168
Size (Mbp)	7.64	7.59	8.00
Contig N50	368.6 kb	349.7 kb	273.8 kb
DNA G+C content	63.5%	64 %	63.5%
No. of CDSs	7052	6915	7427
No. of complete 16S rRNA genes	1	1	1
No. of CRISPRs	2	1	2
Completeness (%)	99.24	99.24	99.01
Contamination (%)	2.34	1.72	1.78

Table S2. Complete pathway modules identified by BlastKOALA analysis in the whole-genome sequences of 1, strain Zidic-5T; 2, *Labrys portucalensis* LMG 23412^{T}; 3, Labrys neptuniae LMG 23578^{T}; 4, *Labrys okinawensis* DSM 18385^{T}.

**Pathway modules**	1	2	3	4
**Carbohydrate metabolism**				
Central carbohydrate metabolism				
Glycolysis (Embden-Meyerhof pathway), glucose => pyruvate	+	+	+	+
Glycolysis, core module involving three-carbon compounds	+	+	+	+
Gluconeogenesis, oxaloacetate => fructose-6P	+	+	+	+
Pyruvate oxidation, pyruvate => acetyl-CoA	+	+	+	+
Citrate cycle (TCA cycle, Krebs cycle)	+	+	+	+
Citrate cycle, first carbon oxidation, oxaloacetate => 2-oxoglutarate	+	+	+	+
Citrate cycle, second carbon oxidation, 2-oxoglutarate => oxaloacetate	+	+	+	+
Pentose phosphate pathway (Pentose phosphate cycle)	+	+	+	+
Pentose phosphate pathway, oxidative phase, glucose 6P => ribulose 5P	+	+	+	+
Pentose phosphate pathway, non-oxidative phase, fructose 6P => ribose 5P	+	+	+	+
Pentose phosphate pathway, archaea, fructose 6P => ribose 5P	-	-	-	+
PRPP biosynthesis, ribose 5P => PRPP	+	+	+	+
Entner-Doudoroff pathway, glucose-6P => glyceraldehyde-3P + pyruvate	+	+	+	-
Other carbohydrate metabolism				
D-Glucuronate degradation, D-glucuronate => pyruvate + D-glyceraldehyde 3P	-	+	-	-
D-galactonate degradation, De Ley-Doudoroff pathway, D-galactonate => glycerate-3P	+	+	+	+
Glycogen biosynthesis, glucose-1P => glycogen/starch	+	+	+	+
Nucleotide sugar biosynthesis, glucose => UDP-glucose	+	+	+	+
UDP-N-acetyl-D-glucosamine biosynthesis, prokaryotes, glucose => UDP-GlcNAc	+	+	+	+
dTDP-L-rhamnose biosynthesis, glucose-1P => dTDP-L-Rha	+	+	+	+
Glyoxylate cycle	+	+	+	+

Propanoyl-CoA metabolism, propanoyl-CoA => succinyl-CoA	+	+	+	+
**Energy metabolism**				
Carbon fixation				
Reductive pentose phosphate cycle (Calvin cycle)	+	+	+	-
CAM (Crassulacean acid metabolism), dark	+	+	+	+
CAM (Crassulacean acid metabolism), light	+	+	+	+
Phosphate acetyltransferase-acetate kinase pathway, acetyl-CoA => acetate	+	+	+	-
Nitrogen metabolism				
Assimilatory nitrate reduction, nitrate => ammonia	+	+	+	+
Sulfur metabolism				
Assimilatory sulfate reduction, sulfate => H2S	+	+	+	+
ATP synthesis				
NADH:quinone oxidoreductase, prokaryotes	+	+	+	+
Succinate dehydrogenase, prokaryotes	+	+	+	+
Cytochrome c oxidase, prokaryotes	+	+	+	+
Cytochrome bd ubiquinol oxidase	+	+	+	+
Cytochrome o ubiquinol oxidase	+	+	+	+
Cytochrome c oxidase, cbb3-type	+	+	+	+
F-type ATPase, prokaryotes and chloroplasts	+	+	+	+
**Lipid metabolism**				
Fatty acid metabolism				
Fatty acid biosynthesis, initiation	+	+	+	+
Fatty acid biosynthesis, elongation	+	+	+	+
beta-Oxidation, acyl-CoA synthesis	+	+	+	+
Lipid metabolism				
Phosphatidylcholine (PC) biosynthesis, PE => PC	+	+	+	+
Phosphatidylethanolamine (PE) biosynthesis, PA => PS => PE	+	+	+	+
**Nucleotide metabolism**				
Purine metabolism				
De novo purine biosynthesis, PRPP + glutamine => IMP	+	+	+	+
Adenine ribonucleotide biosynthesis, IMP => ADP,ATP	+	+	+	+
Guanine ribonucleotide biosynthesis, IMP => GDP,GTP	+	+	+	+
Deoxyribonucleotide biosynthesis, ADP/GDP/CDP/UDP => dATP/dGTP/dCTP/dUTP	+	+	+	-
Adenine ribonucleotide degradation, AMP => Urate	+	+	+	+
Guanine ribonucleotide degradation, GMP => Urate	+	+	+	+

Pyrimidine metabolism				
Pyrimidine ribonucleotide biosynthesis, UMP => UDP/UTP,CDP/CTP	+	+	+	+
Pyrimidine deoxyribonucleotide biosynthesis, UDP => dTTP	+	+	+	-
Pyrimidine degradation, uracil => beta-alanine, thymine => 3-aminoisobutanoate	+	+	+	+
**Amino acid metabolism**				
Serine and threonine metabolism				
Serine biosynthesis, glycerate-3P => serine	+	+	+	+
Threonine biosynthesis, aspartate => homoserine => threonine	+	+	+	+
Glycine cleavage system	+	+	+	+
Betaine biosynthesis, choline => betaine	+	+	+	+
Betaine degradation, bacteria, betaine => pyruvate	+	+	+	+
Ectoine degradation, ectoine => aspartate	+	+	+	-
Cysteine and methionine metabolism				
Cysteine biosynthesis, serine => cysteine	+	+	+	+
Methionine biosynthesis, aspartate => homoserine => methionine	+	+	+	-
Branched-chain amino acid metabolism				
Valine/isoleucine biosynthesis, pyruvate => valine / 2-oxobutanoate => isoleucine	+	+	+	+
Isoleucine biosynthesis, threonine => 2-oxobutanoate => isoleucine	+	+	+	+
Leucine biosynthesis, 2-oxoisovalerate => 2-oxoisocaproate	+	+	+	+
Leucine degradation, leucine => acetoacetate + acetyl-CoA	+	+	+	+
Lysine metabolism				
Lysine biosynthesis, succinyl-DAP pathway, aspartate => lysine	+	+	+	+
Arginine and proline metabolism				
Ornithine biosynthesis, glutamate => ornithine	+	+	+	+
Arginine biosynthesis, ornithine => arginine	+	+	+	+
Proline biosynthesis, glutamate => proline	+	+	+	+
Proline degradation, proline => glutamate	+	+	+	+
Polyamine biosynthesis				
Polyamine biosynthesis, arginine => ornithine => putrescine	+	+	+	+
Histidine metabolism				
Histidine biosynthesis, PRPP => histidine	+	+	+	+

Histidine degradation, histidine => N-formiminoglutamate => glutamate	+	+	+	+
Aromatic amino acid metabolism				
Shikimate pathway, phosphoenolpyruvate + erythrose-4P => chorismate	+	+	+	+
Tryptophan biosynthesis, chorismate => tryptophan	+	+	+	+
Other amino acid metabolism				
Glutathione biosynthesis, glutamate => glutathione	+	+	+	+
**Metabolism of cofactors and vitamins**				
Cofactor and vitamin metabolism				
NAD biosynthesis, aspartate => quinolinate => NAD	+	+	+	+
Coenzyme A biosynthesis, pantothenate => CoA	+	+	+	+
Biotin biosynthesis, pimeloyl-ACP/CoA => biotin	+	+	+	-
Lipoic acid biosynthesis, plants and bacteria, octanoyl-ACP => dihydrolipoyl-E2/H	+	+	+	+
Molybdenum cofactor biosynthesis, GTP => molybdenum cofactor	+	+	+	+
PreQ1 biosynthesis, GTP => 7-Aminomethyl-7-deazaguanine	-	+	+	-
C1-unit interconversion, prokaryotes	+	+	+	+
**Biosynthesis of other secondary metabolites**				
Biosynthesis of other bacterial compounds				
Aerobactin biosynthesis, lysine => aerobactin	+	+	+	-
**Xenobiotics biodegradation**				
Aromatics degradation				
Benzoate degradation, benzoate => catechol / methylbenzoate => methylcatechol	+	+	+	-
Catechol ortho-cleavage, catechol => 3-oxoadipate	+	+	+	-

Table S3. Results of the API 50 CH and API ZYM tests. 1, strain Zidic-5^T; 2, *Labrys portucalensis* LMG 23412^T; 3, *Labrys neptuniae* LMG 23578^T. +, positive reaction; -, negative reaction; W+, weak positive.

**API test results**	**1**	**2**	**3**
API 50 CH			
Glycerol	+	+	+
Erythritol	-	-	+
D-Arabinose	-	-	-
L-Arabinose	+	+	+
D-Ribose	+	+	+
D-Xylse	+	+	+
L-Xylose	-	-	w+
D-Adonithol	+	+	+
Metil-β-D-xylopyranoside	-	-	-
D-Galactose	+	+	+
D-Glucose	+	+	+
D-Fructose	+	+	+
D-Mannose	+	+	+
L-Sorbose	+	+	+
L-Rhamnose	-	-	+
Dulcitol	+	+	+
Inositol	+	+	+
D-Mannitol	+	+	+
D-Sorbitol	-	+	+
Methyl-α-D-mannopyranoside	-	-	-
Methyll-α-D-glucopyranoside	-	-	-
N-Acetyl-D-glucosamine	-	-	-
Amygdalin	-	-	-
Arbutin	-	-	+
Esculine	+	+	+
Salicin	-	-	-
D-Cellobiose	+	+	+
D-Maltose	-	-	+
D-Lactose	-	-	+
D-Melibiose	+	+	+
D-Sacharose	-	-	+
D-Tehalose	-	+	+
Inulin	-	-	-
D-Melezitose	-	-	-
D-Raffinose	-	-	+
Starch	-	-	-
Glycogen	-	-	-
Xylitol	+	+	+
Gentiobiose	+	-	+
D-Turanose	+	-	+
D-Lyxose	+	+	+
D-Tagatose	+	+	+
D-Fucose	+	+	+

L-Fucose	-	-	+
D-Arabitol	+	+	+
L-Arabitol	-	-	+
Potassium-gluconate	+	+	+
Potassium-2-ketogluconate	-	-	-
Potassium-5- ketogluconate	-	-	-
API ZYM			
Alkaline phosphatase	+	+	+
Esterase (C4)	+	+	+
Esterase lipase (C8)	-	-	-
Lipase (C14)	-	-	-
Leucine arylamidase	+	+	+
Valine arylamidase	+	+	+
Cystine arylamidase	-	-	-
Trypsine	-	-	-
α-Chymotrypsine	-	-	-
Acid phosphatase	+	+	+
Naphtol-AS-BI-phosphohydrolase	-	-	+
α-Galactosidase	-	-	-
β-Galactosidase	-	-	-
β-Glucuronidase	-	-	-
α-Glucosidase	-	-	-
β- Glucosidase	-	-	-
N-Acetyl- β-glucosaminidase	-	-	-
α-Mannosidase	-	-	-
α-Fucosidase	-	-	-
